# Defecation Habits in Preschoolers Are Associated with Physical Activity: A Cross-Sectional and Isotemporal Substitution Analysis

**DOI:** 10.3390/children10060951

**Published:** 2023-05-26

**Authors:** Yoshinori Komeno, Tsutomu Kuchiki, Yumiko Onodera, Shuichi Machida

**Affiliations:** 1 Faculty of Health Science, Hyogo University, 2301 Hiraokacho Shinzaike, Kakogawa 675-0195, Hyogo, Japan; kuchiki@hyogo-dai.ac.jp; 2 Graduate School of Health and Sports Science, Juntendo University, 1-1 Hiraka-gakuendai, Inzai 270-1695, Chiba, Japan; machidas@juntendo.ac.jp; 3 Faculty of Sports Sciences, Tokyo Women’s College of Physical Education, 4-30-1 Fujimidai, Kunitachi 186-8668, Tokyo, Japan; 4 Institute of Health and Sports Science & Medicine, Juntendo University, 1-1 Hiraka-gakuendai, Inzai 270-1695, Chiba, Japan

**Keywords:** isotemporal substitution analysis, accelerometer, defecation habits, preschoolers

## Abstract

There is a lack of research on the relationship between defecation habits (DF) and physical activity (PA) in preschoolers. This study aimed to clarify the relationship between sedentary behaviour (SB), PA time, and DF in preschoolers and to estimate the effect of DF replacement in an isotemporal substitution (IS) model. The participants included 166 children (aged 4–6 years) attending childcare facilities. PA was measured using an accelerometer to calculate the daily activity and wearing time for SB, light-intensity physical activity (LPA), and moderate-to-vigorous-intensity physical activity (MVPA). DF were classified based on defecation frequency and timing. A multinomial logistic regression analysis was used for the IS model. One-way analysis of variance detected significant differences in MVPA between the DF groups (F(2) = 3.12, *p* < 0.05). According to the analysis results of the IS model, replacing 5 min of SB with MVPA resulted in improved DF (odds ratio [OR], 0.89; 95% confidence interval [CI], 0.81–0.97). Conversely, replacing 5 min of MVPA with SB worsened DF (OR, 1.13; 95% CI, 1.03–1.23). The findings suggest that PA is associated with DF among preschoolers. It also indicates that replacing SB with MVPA could help improve DF in children.

## 1. Introduction

The prevalence of functional constipation among children aged 3–8 years in Japan has been reported to be 20.0% [1]. For children during their developmental years, being constipated may hinder not only their physical and mental health but also their school life, as they are unable to develop defecation function [2]. Therefore, clinical practice guidelines for paediatric chronic functional constipation, jointly prepared by the Japanese Society for Paediatric Gastroenterology, Hepatology, and Nutrition and the Japanese Society for Paediatric Neuro-gastroenterology, mention that it is preferable for children to develop regular defecation habits (DF) before school age [2,3].

Primary school children who are constipated are less regularly involved in sporting activities than those who are non-constipated [2]. This suggests that physical activity (PA) and/or exercise may be related to the prevention of constipation in children. Seidenfaden et al. [4] suggested that PA affects the likelihood of developing constipation at ages 10–18. However, they conducted the study using the same questionnaire for participants who were 1–18 years old. Preschoolers aged 1–6 may have difficulty self-administering and may lack the accuracy to answer the same questionnaire, suggesting that the reliability of the questionnaire may not be sufficient [4]. Validation studies by Reilly et al. [5] and Pfeiffer et al. [6] showed that the accelerometer is a tool for representative evaluation of exercise intensity for both children and adults.

Sedentary behaviour (SB) and PA have been associated with various health outcomes [7,8]. Driessen et al. [9] reported that PA was associated with a decreased risk of functional constipation in the preschool period. Moreover, Huang et al. [10] have shown that constipation is associated with insufficient PA and excessive SB among Chinese adolescents, with a dose–response relationship. In a study involving more than 60,000 women, it was found that those who engaged in moderate-to-vigorous physical activity (MVPA) daily were 44% less likely to report constipation than those who did less than once weekly [11]. These studies suggest that it is important to evaluate exercise intensity to clarify the relationship between PA and constipation [12,13,14,15].

PA studies are mainstream because of the use of isotemporal substitution (IS) models, which perform statistical analyses based on the composition of time and intensity of daily PA, [16] that is, 24 h in a day to examine a person’s life behaviour, including PA. Sasai et al. [17] pointed out that the interdependence between the duration of PA and SB must be considered. For adults, it seems that reallocation of SB to light-intensity physical activity (LPA) or MVPA is associated with a significant reduction in mortality risk [18], and improvement in health-related quality of life [19]. Similar findings have been noted in PA research on preschoolers. Replacement of PA has been shown to reduce obesity and cardiometabolic risks in preschoolers [20,21,22]. The IS model may provide more useful results when examining the relationship between PA and DF in preschool children. In particular, this analysis can help parents, other caregivers, healthcare practitioners, and school administrators to plan and implement intervention programs to ensure the development for healthy defecation of preschool-aged children.

This study aimed to clarify the relationship between SB, PA, and DF in preschoolers and estimate the effect of SB and PA replacement on DF using an IS model.

## 2. Materials and Methods

### 2.1. Participants and Procedures

The participants were children aged 4–6 years attending nursery schools in Hyogo, Fukuoka, or Kumamoto Prefecture, Japan. Data were collected between October and November of 2018 and 2019. A total of 182 children agreed to participate in this study. Of these, 166 preschoolers met the inclusion criteria and had valid accelerometer wear times and complete outcome data (physical and psychosocial functioning). The parents and guardians of the participants provided written and verbal informed consent for their children’s participation. The study was approved by the ethical review board of Hyogo University (Approval Number: 18009; date of approval: 9 August 2018; Approval Number: 19009; date of approval: 17 July 2019).

### 2.2. Accelerometer

SB and PA were assessed based on movement behaviours while awake using an accelerometer (Active style Pro HJA 750-C; Omron Healthcare, Kyoto, Japan). Participants were asked to wear the accelerometer on their waist for at least 10 days, except when sleeping or during water-based activities (e.g., bathing, showering, and swimming) [23].

This device (74 × 34 × 46 mm; 60 g) measures the anteroposterior (*x*-axis), mediolateral (*y*-axis), and vertical (*z*-axis) acceleration signals. This accelerometer was validated, and its measurement accuracy was comparable to that of the devices widely used by researchers in Western countries [24]. An epoch-length was set at 10 s, and estimated metabolic equivalents (METs) were obtained using developer-provided software. Non-wear time was defined as intervals of 20 consecutive minutes with activity counts below the detection limit [23]. The inclusion criterion for valid wear time was a minimum of four days, including at least one weekend day [25]. A valid day was required to have a minimum wear time of 10 h [25].

Each 10 s epoch was classified as SB (≤1.5 METs), LPA (1.6–2.9 METs), or MVPA (≥3.0 METs) (Hikihara et al., 2014). Minutes spent on each of these behaviours were aggregated per day and averaged over all valid days.

### 2.3. Defecation Habits

Parents completed the original distributed questionnaire, which consisted of the children’s DF. The definition of DF was vague, ranging from self-reported DF to the use of clinical criteria regarding frequency and symptoms of constipation [26,27]. The questionnaire asked about the defecation frequency (1, almost every day; 2, once every 2–3 days; 3, once every 4–5 days; 4, once a week; 5, irregular) and defecation timing (1, about morning; 2, about noon; 3, about night; 4, not decided). DF was scored based on the frequency and timing of defecation (Table 1). Defecation frequency was categorized into two groups based on the response of ‘almost every day’ (every day and non-every day groups), and defecation timing was categorized into three groups based on the response of ‘about morning’ (morning time and non-morning time groups). Additionally, DF was categorized using defecation frequency and timing: DF1, those who defecated every day in the morning; DF2, every day or in the morning; DF3, once every two days and not at a fixed time. In a review of child constipation by Loening-Baucke [28], daily bowel movement was defined as one of the goals of the algorithm for education and treatment of childhood constipation [28,29]. A review article also mentioned that regular defecation habits should be accomplished in childhood constipation management [28,29]. It should be noted that this study limited regular DF to the morning.

### 2.4. Covariates

Participants reported their age (months), sex, and body mass index (BMI). BMI was calculated based on the measured height and weight. These were adjusted for this study, as they are related to PA and SB in previous studies [24,30].

### 2.5. Statistical Analyses

The analyses were conducted using IBM SPSS Statistics 26.0 (IBM Japan Corp., Tokyo, Japan). The Kolmogorov–Smirnov test was used to check the normality of the data.

One-way analysis of variance (ANOVA) and the IS model were used to examine the associations between SB, LPA, and MVPA for each of the three DF groups. A multinomial logistic regression analysis was used for the IS model. We used 5 min as a unit for activity; thus, the IS models examined the effect of replacing 5 min of one activity with the same amount of another activity [31].

The IS model estimates the effect of substituting one activity type with another for the same amount of time (e.g., replacing MVPA with SB by removing SB from the model). It (in the case of omitting SB from the model) was expressed as follows: outcome variable = LPA + MVPA + total wear time (WT) + covariates. In this model, the corresponding coefficients represent the association of replacing the activity removed with the other activity, while keeping all other variables constant. Cross-sectional models were adjusted using WT and covariates.

## 3. Results

The participant characteristics are shown in Table 2. The mean age was 65.7 ± 8.5 months, over half were male (53.6%), and the mean BMI was 15.7 ± 1.3 kg/m^2^. Overall, 26.2% of children defecated once a day in the morning, 45.5% defecated daily or in the morning, and 28.2% had irregular defecation habits. The mean step was 9377 ± 1850, and the mean accelerometer wearing time was 832.4 ± 74.0 min per day. On average, the children spent 39.6% of their monitored time sedentarily. The time spent on LPA and MVPA were 39.0% and 21.4%, respectively.

The results of the one-way ANOVA for the sample are shown in Table 3. ANOVA detected significant differences in steps and MVPA (F(2) = 3.50, *p* < 0.03; F(2) = 3.12, *p* < 0.05). According to Tukey’s HSD test, the DF1 group had more steps and MVPA than the DF3 group (*p* < 0.03; *p* < 0.05). There was no significant difference for WT, SB, and LPA between the groups.

Table 4 presents the results of the IS models for the sample. First, the results for DF1 and DF3 are presented. Replacing 5 min of total sedentary time with MVPA was significantly associated with a lower defecation score. Replacing 5 min of LPA with MVPA yielded the same results. No significant difference was found between the other groups.

## 4. Discussion

We measured the amount of PA using an accelerometer and conducted a questionnaire-based survey on DF among 166 preschool children aged 4–6 years. The main findings of this study demonstrated that DF was associated with daily steps and MVPA. Furthermore, it was found that substituting MVPA for 5 min of SB was significantly associated with DF scores using an IS model. We found new evidence that reallocating time from SB or LPA to MVPA is associated with better DF, reinforcing the importance of adequate PA for managing DF in 4–6-year-old children. These results provide evidence for the association between PA and DF in preschoolers.

This study explored physical activities of different intensity and evaluated their impact on the defecation habits of children. We found that DF in preschoolers was associated with daily steps and MVPA but not with SB and LPA. Several studies have reported a relationship between PA and constipation in children [2,7,32]. Driessen et al. [7] and Huang et al. [8] noted that insufficient PA was associated with constipation in children. However, some reports have demonstrated no association between PA and constipation in children [2,32]. Inan et al. [2] and Chien et al. [32] evaluated the amount of PA using a questionnaire survey and did not measure the exercise intensity. Driessen et al. [7] reported that PA in preschool children was associated with a reduced risk of functional constipation by assessing exercise intensity using accelerometers. This is consistent with our results. Therefore, the use of accelerometers enabled the daily monitoring of children’s PA and provided a more accurate evaluation of exercise intensity.

We replaced 5 min of total sedentary time with MVPA, which was significantly associated with a lower defecation score. This study is the first to examine the associations between PA and DF in preschoolers while exploring the interdependence of these behaviours in a day using the IS model. We found new evidence that reallocating time from SB or LPA to MVPA is associated with better DF, reinforcing the importance of adequate MVPA in managing defecation in preschoolers. Although many epidemiological studies have suggested that engaging in MVPA (e.g., exercise) has beneficial effects on health outcomes [16], this study showed not only that higher PA was associated with DF but also that replacing LPA and SB with MVPA for only 5 min is independently associated with DF in preschool-aged children. SB and LPA were associated with DF only in the IS analysis. Huang et al. [8] reported that constipation in Chinese adolescents was associated with inadequate PA and excessive SB, which is consistent with our results. In contrast, we did not find evidence that the reallocation of time from SB to LPA is associated with DF. Further evidence regarding children was presented by Collings et al. [33] who found that substituting SB with LPA or MVPA was inversely associated with fat mass index and trunk fat mass index. When exploring fitness levels among children, Leppänen et al. [34] found that the reallocation of SB to LPA or MVPA was associated with greater cardiorespiratory fitness. Our findings are inconsistent with these results. The probable reason is that the waist-mounted accelerometer might not be able to fully distinguish between postural sitting and standing [35], resulting in occasional misclassification of SB and LPA. Therefore, in this study, association with DF was emphasized only for replacements including MVPA.

Our study indicated that a good approach for managing defecation was to substitute the time spent in SB or LPA with MVPA. To facilitate this, parents (or other caregivers) and teachers can decrease their screen time, thus lowering SB and LPA in daily life. Children could reduce SB, such as riding in a car during transportation time and playing indoors, to increase the time available for MVPA. However, our research also suggests that replacing MVPA with SB or LPA may worsen DF. In other words, the loss of outdoor playtime that guarantees MVPA due to adult convenience and environmental factors may lead to defecation dysfunction and constipation if replaced with SB or LPA. This demonstrates the importance of ensuring MVPA in the development of defecation function and quality of life in preschoolers.

A valuable finding of this study was that lifestyle habits of defecation were associated with PA in children aged 4–6 years. However, evidence regarding the association between MVPA and DF in children is scarce. Kohyama [36] found that poor defecation frequency was significantly associated with various lifestyle habits such as PA, skipping breakfast, and increased screen time among Japanese children from the fifth grade of elementary school to the third grade of high school. Yamada et al. [29] found that non-daily and totally irregular DF showed significant associations with various lifestyle habits, such as physical inactivity, skipping breakfast, long screen time, and late bedtime among Japanese children aged 9 to 10 years. These results are consistent with our findings. It is desirable for children to acquire DF before school [2,3]. The acquisition of toileting skills, defined as the process of establishing continence, is considered an important milestone in normal childhood development [37,38]. By the age of 4 years, children usually acquire bowel and bladder control [38]. Amano et al. [39] suggested that self-control of defecation is established between 1.5–4 years of age. Interestingly, the age for the onset of functional constipation peaks around 1 year and decreases after 4 years, with a median of 2.3 years [40]. It should be emphasised that early childhood is considered to play an important role in the development of defecation.

The limitations of this study include the fact that the data were collected from a relatively small sample. Therefore, our findings may not be generalisable to the overall Japanese population. Additionally, the small sample size limited covariates. Further research using a larger sample size is needed to better understand the DF of preschool-aged children. Second, the cross-sectional nature of this study limits it to draw causal inferences. Additionally, statistical analysis in the IS model did not include all daily movement-related behaviours (e.g., sleep, SB, LPA, and MVPA) for 24 h. Third, data on frequency and timing of DF were based on subjective responses. Third, the definition of DF was based on subjective parental responses rather than clinical assessment. The use of the Rome criteria for international assessment of constipation and the use of objective assessments such as bowel duration and stool consistency may provide more reliable information about bowel habits. Finally, accelerometer measurements of SB and PA may have limitations in children. Factors such as device placement and subject non-compliance may have affected the accuracy of the measurements. The use of METs as a measure of PA does not capture the full range of physical activity intensity in preschoolers, nor does it consider individual differences in fitness levels. To confirm the causality and validity of our findings, future studies should be conducted with statistical analysis (compositional data analysis [CoDA]) considering all daily movement-related behaviours for 24-h longitudinal data.

## 5. Conclusions

The novelty of this study was in focusing on preschool-aged children, among whom the development of healthy defecation habits was crucial for their physical and mental health, and evaluating the importance of physical activities of different intensity on defecation habits. This study showed that DF in preschoolers was associated with MVPA but not with SB or LPA. Furthermore, this study is the first to examine the associations between PA and DF in preschoolers while accounting for the interdependence of these behaviours in a day using the IS model. Previous studies had only shown that PA may contribute to improving the quality of DF [4,36]. However, our study can be applied to set exercise intensity and time when conducting intervention studies, eventually leading to the realisation of optimal education and management for the development of defecation function in preschool children. On the other hand, our research suggests that replacing MVPA with SB or LPA may worsen DF, although, substitution for inactivity is not possible in intervention studies. This study may demonstrate certain benefits in public health and health guidance for preschool-aged children. These benefits will be reinforced by expanding the findings through further longitudinal and cohort studies.

## Figures and Tables

**Table 1:** Defecation habit score using defecation frequency and timing.

	Frequency	Every Day	Once Every 2–3 Days	Once Every 4–5 Days	About Once a Week	Irregular
Timing						
**Morning**		DF1	DF2			
**Noon**		DF2	DF3			
**Night**						
**Not decided**						

DF1: Defecate every morning, DF2: Defecate every day or morning time, DF3: Defecate once every two days or less.

**Table 2:** Characteristics of participants in this study.

Age, months					65.7 ± 8.5	
Gender, *n* (%)						
	Male				89 (53.6%)	
	Female				77 (46.4%)	
Body mass index, kg/m^2^					15.7 ± 1.3	
Defecation habit, *n* (%)						
	Defecate every morning (DF1)				38 (26.2%)	
	Defecate every day or morning time (DF2)				66 (45.5%)	
	Defecate once every two days or less (DF3)				41 (28.2%)	
Accelerometer data						
	Step, steps				9377 ± 1850	
	Wear time, min/day				832 ± 74.0	
	SB time, min/day				330 ± 71.6	
	LPA time, min/day				325 ± 44.3	
	MVPA time, min/day				179 ± 34.1	
Mean ± SD or number (%)						
*n* = 166						

SB: Sedentary behaviour, LPA: Light-intensity physical activity, MVPA: Moderate-to vigorous-intensity physical activity.

**Table 3:** Test for the difference in accelerometer data for defecation habit.

	Defecation Habit		
	DF1	DF2	DF3
Accelerometer data (Mean ± SD)			
Step, steps	9887 ± 1934	9449 ± 1886	8785 ± 1759 *
Wear time, min/day	823 ± 69.3	841 ± 79.9	834 ± 71.4
SB time, min/day	312 ± 69.1	328 ± 74.2	340 ± 75.6
LPA time, min/day	328 ± 43.6	331 ± 43.8	326 ± 44.3
MVPA time, min/day	187 ± 32.1	182 ± 35.5	169 ± 32.4 *

Methods of statistical analysis: One-way ANOVA with Tukey’s HSD, * *p* < 0.05 vs. DF1, DF1: Defecate every morning, DF2: Defecate every day or morning time, DF3: Defecate once every two days or less, SB: Sedentary behaviour, LPA: Light-intensity physical activity, MVPA: Moderate-to-vigorous-intensity physical activity.

**Table 4:** Associations between the defecation habit score and SB, LPA, and MVPA using isotemporal substitution models.

			Exp.(B)	95% CI	*p*
Inactive	⇒	Active			
SB	⇒	LPA	1.00	(0.83, 1.20)	0.98
SB	⇒	MVPA	0.89	(0.81, 0.97)	<0.01
LPA	⇒	MVPA	0.89	(0.80, 1.00)	0.04
Active	⇒	Inactive			
LPA	⇒	SB	1.00	(0.94, 1.06)	0.98
MVPA	⇒	SB	1.13	(1.03, 1.23)	<0.01
MVPA	⇒	LPA	1.13	(1.00, 1.26)	0.04

Methods of statistical analysis. Multiple logistic regression analysis, Comparison group: DF1(Defecate every morning), DF3 (Defecate once every two days or less). All models were adjusted for gender and body mass index, and replacement is 5 min. SB: Sedentary behaviour, LPA: Light-intensity physical activity, MVPA: Moderate-to vigorous-intensity physical activity.

## Data Availability

Data can be requested from the corresponding author.
